# Are Ancient Durum Wheats Less Toxic to Celiac Patients? A Study of **α**-Gliadin from Graziella Ra and Kamut

**DOI:** 10.1100/2012/837416

**Published:** 2012-05-02

**Authors:** M. Stella Colomba, Armando Gregorini

**Affiliations:** Dipartimento di Scienze della Terra, della Vita e dell'Ambiente (DiSTeVA), Università di Urbino “Carlo Bo”, Via Maggetti 22, 61029 Urbino, Italy

## Abstract

In the present paper, the controversial hypothesis suggesting ancient grains might show lower immunogenic properties and therefore the possibility to introduce them in the diet of wheat-sensitive people, including celiac patients, was investigated. The immunogenic potential of the ancient durum wheats Graziella Ra and Kamut was studied by comparison to the durum accessions Cappelli, Flaminio, Grazia and Svevo. Experiments were carried out with two monoclonal antibodies (mAbs) raised against **α**-gliadin peptides p31–49 and p56–75 (the latter containing the overlapping DQ2-Glia-**α**1 and DQ2-Glia-**α**2 epitopes), toxic for celiac patients. For all accessions, a few **α**-gliadin alleles were also cloned, sequenced and translated into aminoacid sequences. Several aminoacid substitutions or deletions were detected in p31–49, DQ2-Glia-**α**1 and DQ2-Glia-**α**2 epitopes, nevertheless, ELISA constantly showed antibody-antigen positive reactions which led us to suggest that mAbs binding was not apparently affected by polymorphisms. Moreover, a few substitutions were also observed in DQ2-Glia-**α**3 and DQ8-Glia-**α**1 epitopes. Although some DQ2-Glia-**α**1 and DQ2-Glia-**α**2 variants evidenced herein were previously reported to have a diminished or abolished T cell stimulatory capacity, present results cannot confirm that ancient durum wheats would be less CD-toxic. In conclusion, we strongly advice celiac patients from consuming ancient wheats including Graziella Ra or Kamut.

## 1. Introduction

Celiac disease (CD) is an intestinal chronic disorder caused by an intolerance to wheat gluten proteins (gliadins and glutenins) mainly resulting in small-intestinal mucosal injuries and nutrient malabsorption in susceptible individuals [1]. In recent years it has become clear that CD is far more common than previously thought. Several serological screening studies from Europe, South America, Australasia, and the USA have shown that approximately 0.5–2% of these populations suffers from CD. Nevertheless, most affected individuals remain undiagnosed due to an increasingly broad spectrum of clinical presentations [2]. Although they are all characterized by a certain degree of villous atrophy, from a clinical point of view, it is usual to consider three different forms of CD: *major or classic CD* (characterized by the presence of symptoms of frank malabsorption, i.e., diarrhea, steatorrhea, and/or weight loss); *minor subclinical CD* (with no evident symptoms of malabsorption, but rather with minor transient or extraintestinal symptoms); and *Silent CD* (referring to patients without any clinical symptoms at all but positive to serological tests such as antigliadin, antiendomysial, and antitransglutaminase, showing evidence of histological change in the small intestine). Hence, screening for silent or subclinical CD has become of paramount importance, especially considering that a proper diagnosis might prevent and/or reduce the severity of symptoms and ultimately favour a remission of associated diseases.

CD is a multifactorial disorder including both genetic and environmental factors whose relative weight is not yet fully understood. Differences in concordance rates in monozygotic (86%) and dizygotic (20%) twins strongly suggest a relevant influence of genetic factors, of which HLA (Human Leukocyte Antigen) is estimated to contribute for 40–50% to disease development [3, 4]. In particular, while roughly 95% of CD patients carries HLA-DQ2 (DQA1*0501/DQB1*0201), most individuals that are not HLA-DQ2 positive express HLA-DQ8 (DQA1*0301/DQB1*0302). Both HLA-DQ2 and HLA-DQ8 have typical peptide-binding motifs characterized by a preference for hydrophobic and negatively charged aminoacids at specific positions in peptides resulting mostly from gliadins digestion [5, 6], although the celiac toxicity of glutenins becoming increasingly appreciated [7].

According to mobility in lactic acid PAGE (A-PAGE), gliadins can be subdivided into four subfractions: *α*/*β*-gliadins, *γ*-gliadins, and *ω*-gliadins, whereas the glutenins consist of low and high molecular weight (LMW and HMW) glutenins, the latter being particularly important for the baking quality of dough. Gliadins have several unique features that contribute to their immunogenic properties. They are extremely rich in proline (P) and glutamine (Q) and, consequently, highly resistant to proteolytic degradation within the gastrointestinal tract, since gastric and pancreatic enzymes lack postproline-cleaving activities [8]. Additionally, the high-glutamine content makes gliadins a good substrate for tissue transglutaminase (tTG), an enzyme constitutively expressed in the *lamina propria* playing a role in tissue repair. Under physiological conditions, tTG can also convert (during the deamidation process) glutamine into the negatively charged glutamic acid (E), leading to enhanced immunogenicity of the resulting modified peptides, which can preferentially bind to HLA-DQ2 or HLA-DQ8 [9, 10]. Deamidation is most likely a crucial event in the generation of a full-blown gluten-specific T-cell response and concomitant CD development. Many gluten peptides with T-cell stimulatory capacity have been identified in all gliadin fractions and in low and high molecular weight glutenins [11, 12]. In particular, as far as concerns *α*-gliadins, four peptides (with a minimal 9-mer epitope core), DQ2-Glia-*α*1 [P(F/Y)PQPQLPY], DQ2-Glia-*α*2 (PQPQLPYPQ), DQ2-Glia-*α*3 (previously reported as Glia-*α*20, FRPQQPYPQ), and DQ8-Glia-*α*1 (QGSFQPSQQN) are known to provoke a specific T-cell response. In addition to a gluten-specific T-cell activation, there is also activity of the innate immune system, mediated by interleukin 15 (IL15) [13] which may be invoked by gliadin peptides, particularly *α*-gliadin 31–49 (toxic core LGQQQPFPPQQPY) that do not stimulate small intestinal T cells [14] but which cause *in vitro* [15, 16] and *in vivo* celiac toxicity [9].

The only effective treatment available for CD patients is a strict exclusion of gluten from the diet. Detrimental consequences of gluten and/or analogous proteins (present in rye, barley and oats) consumption are well documented, showing that noncompliance to a gluten-free diet is associated with increased risk of anemia, infertility, osteoporosis, and intestinal lymphoma [17]. On the other hand, complying with a gluten-free diet is difficult and affects the patients' quality of life, but a strict diet is critical to reduce morbidity and mortality. New treatment strategies are thus actively pursued. Most of these treatments aim to put the celiac patients on a normal diet and add a drug designed to abolish the T-cell stimulatory capacity of gluten. An alternative possibility would be to improve the celiac diet with food items made from baking-quality wheat that do not contain harmful gluten proteins. In this regard, the existence of thousands *Triticum *accessions has raised the question of whether they are equally toxic for CD patients and promoted attempts to generate (by selective breeding or genetic modifications) wheat (and other cereals) with absent or reduced immunogenicity [18, 19]. Moreover, ancient wheats (most frequently not subjected to major genetic improvements) have been speculated, although without any scientific or clinical evidence, to potentially reduce or absent toxicity and therefore to be better suited to be introduced into the diets of people suffering from intolerances or allergies, including celiac patients.

To test such a controversial hypothesis, the CD-immunogenic properties of *α*-gliadins from two ancient wheats, Graziella Ra; an historic accession of durum wheat showing medium-high nutritional properties [20], and Kamut, considered an ancient relative of durum wheat, have been investigated by a comparative analysis with four durum wheats, including Cappelli (the very first selected Italian strain, therefore considered a traditional wheat) and three modern accessions, Flaminio, Grazia, and Svevo. In particular, as a further step of a previous research aimed at evaluating the genetic diversity of these accessions [21], in the present paper we studied *α*-gliadin peptides p31–49 (LGQQQPFPPQQPYPQPQPF) and p56–75 (LQLQPFPQPQLPYPQPQLPY) by two complementary approaches based on standard proteomic and genomic techniques. ELISA was carried out using two specific monoclonal antibodies (mAbs), PN3 and CDC-5, raised against synthetic peptides equivalent to the aminoacids 31–49 and 56–75, respectively. A-gliadin genes from all the accessions were also cloned and sequenced to detect polymorphisms of the two toxic motifs and search for a possible relationship between sequence variability and monoclonal antibody/epitope binding reaction. Additionally, *α*-gliadin genes sequences analysis provided information on DQ2-Glia-*α*3 and DQ8-Glia-*α*1 epitope variants.

## 2. Materials and Methods

### 2.1. Wheat Accessions and Gliadin Extraction

Graziella Ra is a type of durum wheat characterized by low yield (15–20 quintals per hectare), medium-long cycle, tall size (about 120 cm), and a phenotype very similar to Kamut's with large ears and long aristas [20, 21]. It was brought to Italy at the end of ‘70 s, forgotten for a long time and “rediscovered”, due to its fine pasta-making qualities, a few years ago when it appeared on the market as Graziella Ra. Kamut is a registered trademark of Kamut International, Ltd., used in marketing products made with the homonymous grain (cv. QK-77), the correct subspecies of which is still in dispute. In fact, according to Stallknecht et al. [22], Kamut has been classified, from time to time, as *T. turgidum polonicum*, *T. turgidum turanicum,* or *T. turgidum durum*; more recently, Khlestkina et al. [23] suggested that Kamut could be a natural hybrid between *T. durum *and* T. polonicum*. Although its taxonomy is quite contentious, it is generally considered an ancient relative of durum subspecies. Cappelli is an Italian traditional strain of durum wheat which deserves a privileged place among the varieties of old established durum wheat for being the very first selected variety. Svevo, Grazia, and Flaminio are modern durum cultivars, with a great commercial importance, employed for pasta or bread making. All wheats were provided by Alce Nero Cooperative (Isola del Piano, PU, Italy) with the exception of Kamut, kindly supplied by Molini del Conero (Osimo, AN, Italy).

For each accession, wheat kernels were ground in an electric grinder to produce a homogenous sample powder. Subsequently, gliadins were extracted following standard protocols: briefly, 1 g of powder was transferred to 10 mL of 60% (v/v) ethanol for 30 min, shaking vigorously. After centrifugation (3,000 g × 15 min) at room temperature (RT), the supernatant was recovered and stored frozen (−20°C). Whole gliadin from Sigma-Aldrich (G3375) was employed as standard.

### 2.2. Enzyme-Linked Immunosorbent Assay (ELISA)

For each accession, total gliadin was assessed by a commercial two immunological step sandwich assay type, the Gliadin ELISA kit (Immunotech) accordingly to the manufacturer's instructions. Subsequently, *α*-gliadin was determined by indirect two-steps ELISA with PN3 [24] and CDC-5 [25] mAbs following standard procedures [26]. Briefly, 96-microwells plates were coated with samples (diluted 1 : 3,000) and standards (Sigma gliadin at concentrations ranging from 0 ng/mL to 1,000 ng/mL) overnight at 4°C in the dark, incubated with murine mAbs (PN3, diluted 1 : 1,000 or CDC-5, diluted 1 : 2,000) for one hour and goat anti-mouse immunoglobulin (IgG, H + L) conjugated to horseradish peroxidase (Pierce) diluted 1 : 5,000, for one hour. The substrate, tetramethylbenzidine (TMB), was added to plates and after 15 minutes at RT in the dark, absorbance was determined at 450 nm. Results were read off a semilogarithmic calibration curve. Statistical analysis was performed by the one-way ANOVA test, followed by the Bonferroni *post hoc* test.

### 2.3. DNA Extraction, Amplification, Cloning, and Sequencing

Fifteen seeds of each cultivar were germinated in the dark for two days. The seedlings were grown in daylight for seven days. The leaf tissues, sampled at the four-leaf stage from ten different plants per accession, were immediately frozen in liquid nitrogen and ground in a mortar with a pestle. Fifty mg of powder was used for DNA extraction following the cetyltrimethylammonium bromide (CTAB) buffer protocol [27] with slight modifications.

Forward (5′-ATGAAGACCTTTCTCATCC-3′) and reverse (5′-YYAGTTRGTACC GAAGATGCC-3′) primers to amplify *α*-gliadin genes from genomic DNA were designed on the conserved sequences at the 5′ and 3′ ends of the coding region of a few *α*-gliadin gene complete sequences retrieved from the GenBank database. PCR amplification was carried out using a high-fidelity Pfu DNA Polymerase (Promega) as follows: 95°C for 2 min; 95°C for 1 min, 60°C for 30 sec, 72°C for 2 min (30 cycles); 72°C for 5 min. An aliquot (1 *μ*L) of the PCR product was inserted into a pCR 4-TOPO vector by the TA-cloning system and transformation was performed on *Escherichia coli* TOP10 cells following the manufacturer's instructions (Invitrogen). The selected transformants were analysed for presence and correct orientation of the insert by PCR, grown in LB medium overnight and purified by the Wizard Plus SV minipreps kit (Promega). Sequencing of plasmid inserts was done by using automated DNA sequencers at Eurofins MWG Operon (Germany). Sequences were visualized with BioEdit Sequence Alignment Editor version 7.0.5.3 [28], aligned with the ClustalW option included in this software and double checked by eye. Deduced aminoacid sequences were obtained and analysed by BioEdit-dedicated options.

## 3. Results

### 3.1. ELISA

**Figure 1:**
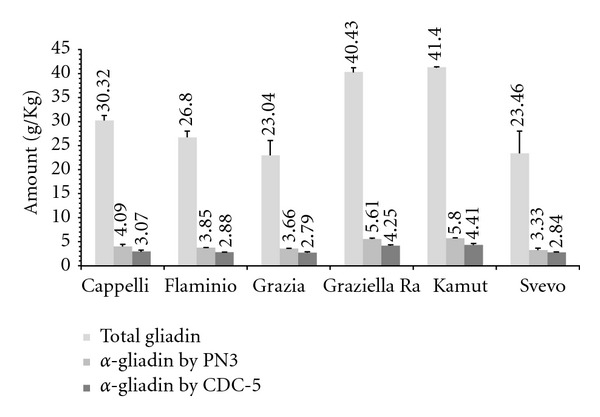
Total gliadin and *α*-gliadin determination by indirect ELISA. Total gliadin was assessed by a commercial kit (Immunotech); *α*-gliadin was evaluated using anti-p31–49 (PN3) and anti-p56–75 (CDC-5) mAbs. Results were read off a semilogarithmic calibration curve, constructed as the dependence of measured absorbance values (vertical axis—linear scale) of corresponding calibrators (standard Sigma—gliadin), range 0–1,000 ng/mL (horizontal axis-logarithmic scale); values are reported as mean ± SD from three independent experiments.

Kamut (K) (41.40 ± 0.10 g/Kg) and Graziella Ra (Gll) (40.43 ± 0.87 g/Kg) had the greater amounts of total (*α*/*β*, *γ* and *ω*) gliadin, followed by Cappelli (C) (30.32 ± 1.06 g/Kg), Flaminio (F) (26.80 ± 1.30 g/Kg), Svevo (S) (23.46 ± 4.67 g/Kg), and Grazia (Gr) (23.04 ± 3.12 g/Kg) (Figure 1). One-way ANOVA showed means to be significantly different (*P* < 0.001), whereas the Bonferroni *post hoc* test determined that Graziella Ra and Kamut were significantly different from Flaminio (*P* < 0.05), Grazia, and Svevo (*P* < 0.01) (Table 1).

For *α*-gliadin assayed using the PN3 (C, 4.09 ± 0.41 g/Kg; F, 3.85 ± 0.02 g/Kg; Gr, 3.66 ± 0.05 g/Kg; Gll, 5.61 ± 0.19 g/Kg; K, 5.80 ± 0.07 g/Kg; S, 3.33 ± 0.39 g/Kg) and CDC-5 (C, 3.07 ± 0.25 g/Kg; F, 2.88 ± 0.03 g/Kg; Gr, 2.79 ± 0.20 g/Kg; Gll, 4.25 ± 0.16 g/Kg; K, 4.41 ± 0.30 g/Kg; S, 2.84 ± 0.12 g/Kg) mAbs (Figure 1) one-way ANOVA showed differences among accessions to be statistically significant (*P* < 0.001); results of Bonferroni correction are reported in Table 1. All experiments gave similar outcomes for both mAbs showing an antibody-antigen-positive reaction in every single case. Hence, according to mABs reactivity, all grains under study, including the two ancient wheats, have high potential immunogenicity and toxicity.

### 3.2. A-Gliadin Gene Sequences

A-gliadin genes from all the accessions were cloned and sequenced. As already known and in line with the relatively high-copy number of *α*-gliadin alleles reported for diploid wheat ancestors (*T. speltoides* and *T. monococcum*) and hexaploid (*T. aestivum*) species [29], in this study as well, several alleles, identified as short (S) or long (L), were characterized differing from each other, at least in this case, mostly in the length of few CAA-rich regions. S alleles ranged from 828 bp to 867 bp, while L alleles were between 894 bp and 963 bp. Every accession showed at least one allele of each type. Moreover, consistent percentages of *α*-gliadin alleles containing one or more internal stop codons (C→T substitution was the most common single-base change observed) were encountered. In line with other studies, we refer to them as pseudogenes, although we cannot predict from the genomic data whether a subset is being expressed [30]. Such findings agree with previous observations that at least 50% of the *α*-gliadin genes is pseudogenes [29, 31].

**Figure 2:**
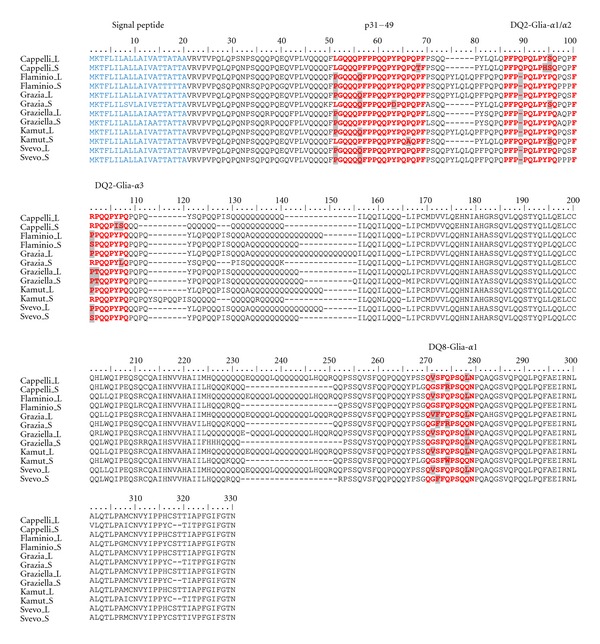
Alignment of deduced L and S *α*-gliadin isoforms of each wheat accession under study. The five epitopes known to be involved in CD, p31–49 (LGQQQPFPPQQPYPQPQPF), DQ2-Glia-*α*1 [P(F/Y)PQPQLPY], DQ2-Glia-*α*2 (PQPQLPYPQ), DQ2-Glia-*α*3 (FRPQQPYPQ), and DQ8-Glia-*α*1 (QGSFQPSQQN) are shown in red. In light grey: aminoacid substitution; hyphen and light grey: aminoacid deletion. For each *α*-gliadin isoform the signal peptide is in light blue.

All sequences were submitted to GenBank database (GQ999806–GQ999831). Each allele was translated (with a BioEdit dedicated option) into the corresponding protein. Deduced aminoacid sequences showed a high degree of similarity (about 75% and 86% for S and L isoforms, resp.). Protein alignment displayed the two epitope motifs (p31–49 and p56–75) in all aminoacid sequences, although with a large series of variants (Table 2; Figure 2). Polymorphisms mainly included one or two aminoacid substitutions (in p31–49, DQ2-Glia-*α*2, DQ2-Glia-*α*3 and DQ8-Glia-*α*1) and a deletion of Q at p_2_ (in DQ2-Glia-*α*2) or at p_4_ (in DQ2-Glia-*α*1). Namely, (1) p31–49 variants included L_1_→P, P_6_→Q, Y_13_→D, P_16_→A, and P_18_→T; (2) DQ2-Glia-*α*1 variants included a deletion of Q at position 4 (observed only in epitopes from locus *Gli-B2*) and the substitution Y_9_→H; (3) DQ2-Glia-*α*2 variants included the substitution P_8_→S (all in epitopes from locus *Gli-A2*), Y_7_→H and a deletion of Q at position 2, epitopes showing the deletion at p_2_ were from locus *Gli-B2*; (4) DQ2-Glia-*α*3 variants included R_2_→P or R_2_→S, P_3_→T, Y_7_→I and P_8_→S or P_8_→L; (5) DQ8-Glia-*α*1 variants included the aminoacid substitutions G_2_→V, S_3_→F, Q_5_→W or Q_5_→R, and Q_9_→L.

## 4. Discussion

Celiac disease, a prototype of T-cell-mediated diseases, is caused by a combination of adaptive and innate immune responses. Generally speaking, T (and B) cells are part of the adaptive immune system, which is characterized by the ability to recognize and remember previous specific antigenic pathogens and adapt its response with time. T cells recognize antigens in the context of MHC molecules, normally displayed by a set of specialized cells called antigen-presenting cells. However, the mere recognition of the MHC-antigen complex is not sufficient to induce a protective T-cell immune response; in fact, T cells have to recognize antigen in the context of an activated innate immune system (i.e., the nonspecific immune system and first line of defense). In celiac disease, gliadin acts as an antigen recognized by the CD4^+^ T cells and, moreover, has the ability to induce an activation of the innate immune response as well [16]. Hence, although the adaptive immune system is central to the development of celiac disease, adaptive immune responses are, however, controlled by an earlier activation of the innate immune system triggered by a fragment of gliadin. Key steps underlying the intestinal inflammatory response to CD include (1) a direct response of the epithelium via the innate immune system to toxic proteins in wheat gluten, (2) modification of wheat gluten proteins by tissue transglutaminase, (3) the role of HLA-DQ2 and HLA-DQ8 in presenting toxic wheat proteins to T cells, and (4) activation of T and B cells [1].

Several T-cell stimulatory *α*-gliadin peptides have been identified, DQ2-Glia-*α*1 [P(F/Y)PQPQLPY], DQ2-Glia-*α*2 (PQPQLPYPQ), DQ2-Glia-*α*3 (FRPQQPYPQ), and DQ8-Glia-*α*1 (QGSFQPSQQN), showing a large diversity of natural variants, many of which have T-cell stimulatory activity [32]. An additional *α*-gliadin peptide, known as p31–49, has been reported to activate the innate immune system by playing an important role as danger signal. It is not the target of gluten-specific T cells but does induce changes associated with CD on administration *in vivo* and during biopsy challenges *in vitro*. It has also been shown that preincubation of biopsy specimens of CD patients with the 31–49 peptide enabled T-cell activation. These effects were found to correlate with the induction of IL-15, a cytokine that is crucial for the activation and survival of memory T cells and induces epithelial changes. Moreover, IL-15 production by enterocytes could have an effect on the adaptive immune response to gluten [13].

In the present study, we characterized *α*-gliadin from Graziella Ra and Kamut in order to investigate (by molecular analyses and inspection of translated aminoacid sequences) the hypothesis suggesting that ancient grains might show lower immunogenic properties and therefore the possibility to introduce them in the diet of wheat-sensitive people, including celiac patients. To this aim, *α*-gliadin main toxic peptides related to CD, p31–49 (LGQQQPFPPQQPYPQPQPF) and p56–75 (LQLQPFPQPQLPYPQPQLPY), were analysed in two ancient durum wheats and in four durum varieties (Cappelli, Flaminio, Grazia, and Svevo) by two different approaches. A first level of analysis was performed by ELISA to evaluate wheat toxicity by specific mAbs raised against the immune-reactive peptides. Subsequently, aminoacid mutations (substitutions and/or deletions) in the toxic motifs, including DQ2-Glia-*α*3 and DQ8-Glia-*α*1, were examined.

By using a commercially available gluten test kit, we determined that Graziella Ra and Kamut have relatively greater amount of gliadin than Cappelli, Grazia, Flaminio, and Svevo. Taking into account that gluten, about 80% of the entire protein reservoir in wheat, is composed by gliadins and glutenins, present results would validate the producers' claims that ancient wheats are endowed with kernels usually bigger and richer in proteins than modern wheats. Interestingly, Cappelli showed an amount of gliadin that is in-between ancient and modern accessions, which supports the placing it as a traditional wheat. When employing a two-step indirect ELISA with PN3 and CDC-5 mAbs, p31–49 and p56–75 were observed in all accessions. In particular, Graziella Ra and Kamut showed the highest values (expressed as *α*-gladin amount), thus challenging the “low-immunogenicity” hypothesis. These findings point out that, in our study, ancient wheats have greater amounts of both total and *α*-gliadin than modern accessions; moreover, taking into account that *α*-gliadins from ancient wheats showed a strong and specific binding reaction to anti-p31–49 and anti-p56–75 mAbs, these accessions may be considered, at least, as toxic as modern ones.

A large series of *α*-gliadin epitope variants, mainly consisting of one or two aminoacid substitutions (Table 2) were detected in all the accessions (including ancient ones). Although their T-cell stimulatory capacity would need to be further investigated, nevertheless, the immunogenic properties of (at least) some of them may be discussed in the light of recently published data. In our study, in fact, we did encounter several variants which, according to Mitea et al. [32], have a diminished or abolished T-cell stimulatory capacity. These include (1) aminoacid deletion at p_4_ in DQ2-Glia-*α*1 (Flaminio, Grazia, Graziella Ra, Kamut, and Svevo); (2) a single substitution of the proline for a serine residue (P→S) at p_8_ in DQ2-Glia-*α*2 (Cappelli, Grazia and Kamut); (3) an arginine to proline substitution (R→P) at p_2_ in DQ2-Glia-*α*3 (Flaminio, Grazia, Graziella Ra, Kamut and Svevo); (4) a single serine to phenylalanine substitution (S→F) at p_3_ (Grazia and Svevo_S); a single glutamine to arginine substitution (Q→R) at p_5_ (Cappelli, Grazia) in DQ8-Glia-*α*1. As far as concerns the other epitope variants reported in the present paper, several have never been described before while some have been described but never tested for their T-cell stimulatory capacity. Granted that accessions under study would deserve a deeper investigation to verify and evaluate their immunogenic capacity, on the other hand, ELISA results (with PN3 and CDC-5 mAbs) demonstrated that detected polymorphisms do not (or little) seem to affect the binding of the monoclonal antibodies to their targets. In fact, both mAbs gave intense positive reactions for all wheats (including ancient ones). Such a finding might be due to the fact that these variants are not contained within the motif recognized by the mAb or, alternatively, although being inside the region, they do not significantly influence the binding reaction. For instance, PN3, which has been shown to bind in the region QQQPFP of the peptide p31–49 [24], in the present study apparently reacted to the QQQQFP variant as well. As far as concerns the effect of DQ2-Glia-*α*1 and DQ2-Glia-*α*2 variants on CDC-5 binding reaction, the issue remains unclear since, unfortunately, the exact sequence within the T-cell stimulating peptide p56–75 identified by the antibody CDC-5 is not known at present. Further studies will be necessary to address this question and evaluate how sensitive CDC-5 is in discriminating DQ2-Glia-*α*1 and DQ2-Glia-*α*2 variants. Although, at the moment we can provide the reader with indirect evidence on the T-cell stimulatory capacity of Cappelli, Flaminio, Grazia, Svevo, Graziella Ra, and Kamut *α*-gliadins, however, even if the above discussed variants were confirmed, by T-cell proliferation assays, to have weak (or absent) T-cell stimulatory properties, all wheats herein examined would still not be safe for CD patients since, besides *α*-gliadins, many other gluten proteins (i.e., *γ*- and *ω*-gliadins, HMW and LMW glutenins) contain stimulatory peptides (relevant to CD pathogenesis) recognized by a heterogeneous repertoire of intestinal T-cell responses [33].

In conclusion, our results demonstrate that (1) the ancient wheats Graziella Ra and Kamut have greater amounts of total and *α*-gliadin than modern accessions; (2) *α*-gliadins from such ancient wheats show a strong and specific binding reaction to anti-p31–49 and anti-p56–75 mAbs; (3) the (putatively) less toxic variants of p31–49, DQ2-Glia-*α*1 and DQ2-Glia-*α*2 epitopes detected in all accessions, including ancient wheats, do not seem to affect mAbs/epitope binding reactions. Therefore, we suggest that Graziella Ra and Kamut are potentially as toxic as modern wheats with reference to CD and strongly recommend that they should not be introduced in the diet of celiac patients.

## Figures and Tables

**Table 1:** Results of Bonferroni multiple comparison *post hoc* test.

Accessions pairwise comparisons	Statistical significance of means differences for total gliadin	Statistical significance of means differences for *α*-gliadin by PN3	Statistical significance of means differences for *α*-gliadin by CDC-5
Cappelli versus Flaminio	ns	ns	***
Cappelli versus Grazia	ns	ns	***
Cappelli versus Graziella	ns	*	*
Cappelli versus Kamut	ns	**	***
Cappelli versus Svevo	ns	ns	***
Flaminio versus Grazia	ns	ns	ns
Flaminio versus Graziella	*	**	**
Flaminio versus Kamut	*	**	ns
Flaminio versus Svevo	ns	ns	***
Grazia versus Graziella	**	**	ns
Grazia versus Kamut	**	**	ns
Grazia versus Svevo	ns	ns	***
Graziella versus Kamut	ns	ns	*
Graziella versus Svevo	**	**	***
Kamut versus Svevo	**	***	***

Bonferroni method (one of the most common used *post hoc* test) was employed to compute the *P* values when comparing pairwise differences of mean values for total gliadin (assessed by a commercial ELISA assay) and *α*-gliadin (assayed using PN3 and CDC-5 mAbs) amounts. ns, NOT SIGNIFICANT;  *P < 0.05;  **P < 0.01;  ***P < 0.001.

**Table 2:** p31–49, DQ2-Glia-*α*1, DQ2-Glia-*α*2, DQ2-Glia-*α*3, and DQ8-Glia-*α*1 epitopes variants detected in durum wheats under study.

Accession	Locus	p31–49	DQ2-Glia-*α*1	DQ2-Glia-*α*2	DQ2-Glia-*α*3	DQ8-Glia-*α*1
Cappelli_L	*Gli-A2*	cs	cs	PQPQLPY**S**Q	cs	Q**V**SFQPSQ**L**N
Cappelli_S	*Gli-A2*	LGQQQPFPPQQPYPQPQ**T**F	PFPQPQLP**H**	PQPQLP**HS**Q	FRPQQP**IS**Q	QGSF**R**PSQQN

Flaminio_L	*Gli-B2*	**P**GQQQ**Q**FPPQQPYPQPQPF	PFP-PQLPY	P-PQLPYPQ	F**P**PQQPYPQ	Q**V**SFQPSQ**L**N
Flaminio_S	*Gli-B2*	**P**GQQQPFPPQQPYPQPQPF	PFP-PQLPY	P-PQLPYPQ	F**S**PQQPYPQ	cs

Grazia_L	*Gli-B2*	**P**GQQQ**Q**FPPQQPYPQPQPF	PFP-PQLPY	P-PQLPYPQ	F**P**PQQPYPQ	Q**VF**FQPSQ**L**N
Grazia_S	*Gli-A2*	LGQQQ**Q**FPPQQP**D**PQPQPF	cs	PQPQLPY**S**Q	FRPQQPY**L**Q	QG**F**F**R**PSQQN

Graziella_L	*Gli-B2*	**P**GQQQPFPPQQPYPQPQPF	PFP-PQLPY	P-PQLPYPQ	F**PT**QQPYPQ	Q**V**SFQPSQ**L**N
Graziella_S	*Gli-B2*	**P**GQQQPFPPQQPYPQPQPF	PFP-PQLPY	P-PQLPYPQ	F**PT**QQPYPQ	QGSFQPSQ**L**N

Kamut_L	*Gli-B2*	**P**GQQQ**Q**FPPQQPYPQPQPF	PFP-PQLPY	P-PQLPYPQ	F**P**PQQPYPQ	Q**V**SFQPSQ**L**N
Kamut_S	*Gli-A2*	LGQQQPFPPQQPYPQ**A**QPF	cs	PQPQLPY**S**Q	cs	QGSF**W**PSQQN

Svevo_L	*Gli-B2*	**P**GQQQ**Q**FPPQQPYPQPQPF	PFP-PQLPY	P-PQLPYPQ	F**P**PQQPYPQ	Q**V**SFQPSQ**L**N
Svevo_S	*Gli-B2*	**P**GQQQ**Q**FPPQQPYPQPQPF	PFP-PQLPY	P-PQLPYPQ	F**S**PQQPYPQ	QG**F**FQPSQQN

Canonical sequences (cs), p31–49: L_1_G_2_Q_3_Q_4_Q_5_P_6_F_7_P_8_P_9_Q_10_Q_11_P_12_Y_13_P_14_Q_15_P_16_Q_17_P_18_F_19_; DQ2-Glia-*α*1: P_1_(F/Y)_2_P_3_Q_4_P_5_Q_6_L_7_P_8_Y_9_; DQ2-Glia-*α*2: P_1_Q_2_P_3_Q_4_L_5_P_6_Y_7_P_8_Q_9_; DQ2-Glia-*α*3: F_1_R_2_P_3_Q_4_Q_5_P_6_Y_7_P_8_Q_9_; DQ8-Glia-*α*1: Q_1_G_2_S_3_F_4_Q_5_P_6_S_7_Q_8_Q_9_N_10_. In bold underlined: aminoacid substitution; hyphen: aminoacid deletion.
